# CSN8 is a key regulator in hypoxia-induced epithelial–mesenchymal transition and dormancy of colorectal cancer cells

**DOI:** 10.1186/s12943-020-01285-4

**Published:** 2020-12-01

**Authors:** Songwen Ju, Feng Wang, Yirong Wang, Songguang Ju

**Affiliations:** 1 Central Laboratory, Affiliated Suzhou Hospital of Nanjing Medical University, Suzhou Municipal Hospital, Suzhou, 215002 Jiangsu Province China; 2 Department of Pathology, Affiliated Suzhou Hospital of Nanjing Medical University, Suzhou Municipal Hospital, Suzhou, 215002 Jiangsu Province China; 3 Department of Immunology, School of Biology and Basic Medical Sciences, Medical College, Soochow University, Suzhou, 215123 Jiangsu Province China; 4 Medical Biotechnology Institute, Soochow University, Suzhou, 215123 Jiangsu Province China; 5 Collaborative Innovation Center of Clinical Immunology between Soochow University and Sihong People’s Hospital, Soochow University, Suzhou, 215123 Jiangsu Province China

**Keywords:** CSN8, Hypoxia, Epithelial–mesenchymal transition, Dormancy, Colorectal cancer

## Abstract

Hypoxic stress plays a pivotal role in cancer progression; however, how hypoxia drives tumors to become more aggressive or metastatic and adaptive to adverse environmental stress is still poorly understood. In this study, we revealed that CSN8 might be a key regulatory switch controlling hypoxia-induced malignant tumor progression. We demonstrated that the expression of CSN8 increased significantly in colorectal cancerous tissues, which was correlated with lymph node metastasis and predicted poor patient survival. CSN8 overexpression induces the epithelial-mesenchymal transition (EMT) process in colorectal cancer cells, increasing migration and invasion. CSN8 overexpression arrested cell proliferation, upregulated key dormancy marker (NR2F1, DEC2, p27) and hypoxia response genes (*HIF-1α, GLUT1*), and dramatically enhanced survival under hypoxia, serum deprivation, or chemo-drug 5-fluorouracil treatment conditions. In particular, silenced CSN8 blocks the EMT and dormancy processes induced by the hypoxia of 1% O_2_ in vitro and undermines the adaptive capacity of colorectal cancer cells in vivo. The further study showed that CSN8 regulated EMT and dormancy partly by activating the HIF-1α signaling pathway, which increased HIF-1α mRNA expression by activating NF-κB and stabilized the HIF-1α protein via HIF-1α de-ubiquitination. Taken together, CSN8 endows primary colorectal cancer cells with highly aggressive/metastatic and adaptive capacities through regulating both EMT and dormancy induced by hypoxia. CSN8 could serve as a novel prognostic biomarker for colorectal cancer and would be an ideal target of disseminated dormant cell elimination and tumor metastasis, recurrence, and chemoresistance prevention.

**Supplementary Information:**

The online version contains supplementary material available at 10.1186/s12943-020-01285-4.

## Main text

Cancer fatalities result from metastasis and therapy resistance, with both processes depending on signals from the tumor microenvironment [1]. Hypoxia is a common feature of the cancer microenvironment and is strongly associated with invasion, metastasis, resistance to therapy, and poor clinical outcomes. Hypoxia plays a crucial role in triggering the epithelial-mesenchymal transition (EMT) by regulating hypoxia-inducible factors (HIFs) [2]. EMT represents a key step toward cancer cell migration from the primary tumor to the distant organs and, ultimately, to disseminated tumor cells (DTCs) [3]. Furthermore, hypoxia has been shown to induce molecular changes within cancer cells to facilitate dormancy, which is a state of “temporary mitotic arrest” [4]. Accumulating evidences indicate that dormancy is essential for cancer cell survival in “hostile” microenvironments at distant sites, as cells become resistant to cancer therapy and evade attack by immune cells [4, 5]. However, the underlying mechanism through which cancer cells undergo EMT, dormancy progression, and evade apoptosis/necrosis under hypoxic environments still remains to be elucidated.

The COP9 signalosome (CSN) is an evolutionarily conserved protein complex composed of 8 subunits (CSN1–CSN8) in higher eukaryotes, and plays an important role in ubiquitin-mediated protein degradation [6]. Previous reports indicated that the subunit of CSN, such as CSN6, is involved the malignant process of cancer [7]. CSN8, the smallest and least conserved subunit among the eight subunits of CSN, is essential for peripheral T cell homeostasis and cardiomyocytes survival [6]. However, the role of CSN8 in cancer biology is still unclear. Here, we demonstrated that the expression of CSN8 is upregulated in colorectal cancer (CRC) tissues and correlates with lymph node metastasis and poor prognosis. CSN8 serves as a key regulator that controls EMT and dormancy induced by hypoxia, partly through modulating the HIF-1α signaling pathway, endowing CRC cells with stronger invasion and metastatic abilities, as well as highly adaptive capacities in response to environmental stress.

## Results and discussion

### CSN8 is upregulated in CRC tissues, and involved in the EMT and dormancy of CRC cells

**Fig. 1:**
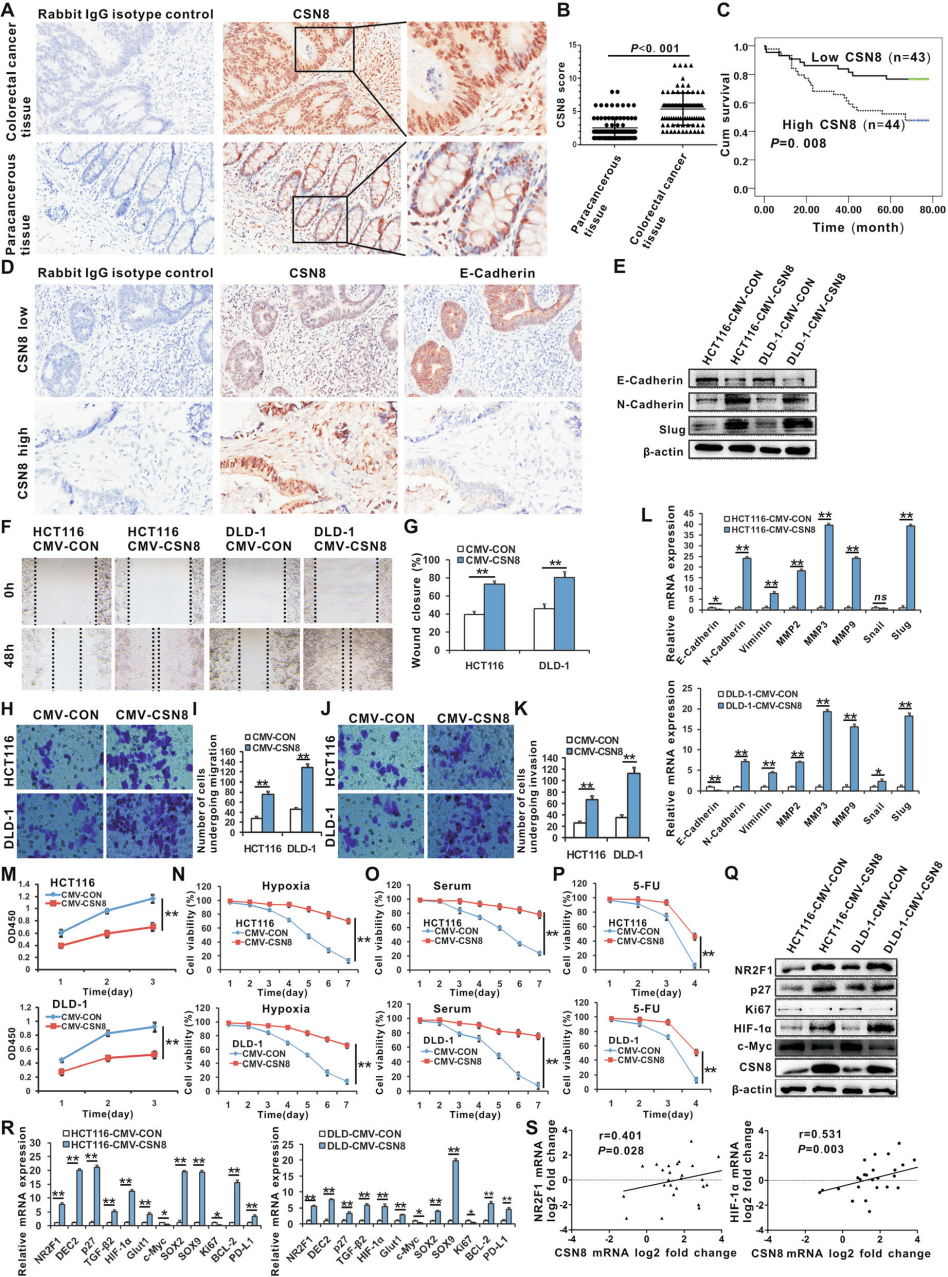
CSN8 expression is upregulated in CRC tissues, and is involved in the EMT and dormancy of CRC cells. **a** Tissue microarray assays analyzed the expression of CSN8 in CRC tissues. Representative immunohistochemistry images showed the expression and subcellular distribution of CSN8 in tumor tissues or tumor-adjacent tissues. **b** Nuclear CSN8 expression is higher in tumor tissues than in tumor-adjacent tissues. **c** Kaplan–Meier survival analysis was conducted according to the CSN8 levels in CRC patients (log-rank test). **d** Representative immunohistochemistry images show the expression of CSN8 and E-Cadherin in tumor tissues. **e** Western-blot analysis of protein levels of E-Cadherin, N-Cadherin, and Slug in CSN8-overexpressed CRC cells and control cells. **f**, **g** Representative scratch-wound images showing the healing ability of CSN8-overexpressed CRC cells and control cells. **h**, **i** The migration ability of CSN8-overexpressed CRC cells and control cells was determined by Transwell migration assay. **j**, **k** The invasive ability of CSN8-overexpressed CRC cells and control cells was analyzed by Matrigel invasion assay. **l** Real-time PCR was used to analyze the mRNA expression of EMT-associated genes. **m** CCK-8 assay analyzed the proliferation activity of CSN8-overexpressed CRC cells and control cells. **n**–**p** Trypan blue exclusion assay was used to analyze the cell viability of CSN8-overexpressed CRC cells and control cells cultured under 20% O_2_ or 1% O_2_, serum deprivation, or 5-FU (20 μg/mL) conditions. **q** Western blot analysis of the protein levels of dormancy-associated genes. **r** Real-time PCR was used to analyze the mRNA expression of dormancy-associated genes. **s** Human CRC tissue cDNA arrays showing changes in CSN8, NR2F1, or HIF-1α transcript expression. Data are presented as the mean ± standard deviation.^*^P < 0.05; ^**^P < 0.01; *ns*, *P ≥* 0.05

To investigate the expression of CSN8 in CRC tissues, we conducted immunohistochemistry (IHC) staining for CSN8 on a CRC tissue microarray (product number: HCol-Ade 180Sur-14) containing 90 pairs of CRC specimens and the corresponding tumor-adjacent tissues. Three pairs of tumor and tumor-adjacent tissues were excluded as they were severely broken, and the samples from the other 87 CRC patients were further analyzed. CSN8 was dominantly expressed in the nucleus and weakly in the cytoplasm of CRC tissues and adjacent tissues (Fig. 1a). CSN8 was expressed at statistically significantly higher levels in the nucleus of CRC tissues, as compared to adjacent tissues (Fig. 1b). The high expression of CSN8 was found to be significantly associated with lymph node metastasis, tumor stage (Additional file 2: Table S1), and poor patient survival (Fig. 1c). The data from the other set of CRC tissue microarray (product number: HCol-Ade 180Sur-06) confirmed the above findings and indicated that there was no significant correlation between the expression of CSN8 and the status of mismatch repair (MMR) proteins (Additional File 3. Fig. S3; Additional File 2. Table S4).

Since EMT is a process that is associated with tumor metastasis and poor prognosis, we then clarified whether the high expression of CSN8 was involved in the EMT process. The serial sections of tissue microarrays by immunohistochemistry (IHC) staining revealed that CSN8 expression was negatively correlated with EMT maker E-Cadherin expression (Fig. 1d; Additional file 2: Table S2). Accordingly, CMV-driven-CSN8 overexpression suppresses the expression of E-Cadherin, whereas it enhances the expression of mesenchymal marker genes (N-Cadherin and Vimentin), the EMT transcription factor (EMT-TF) Slug, and matrix metallopeptidases (MMP2, MMP3 and MMP9) in CRC cell lines HCT116 and DLD-1 (Fig. 1e and l). Wound-healing assays showed that CSN8 overexpressed HCT116, and that DLD-1 cells healed the wound much faster than did the control cells (Fig. 1f and g). Transwell migration assay (Fig. 1h and i) and the matrigel invasion assay (Fig. 1j and k) showed that the over-expression of CSN8 increased the migration and invasion of CRC cell lines.

Since dormancy, a state of permanent growth arrest, endowed cancer cells’ strong resistance and tolerance to environmental stress, we discussed the relationship between CSN8 and the dormancy of CRC cells. As expected, the CCK-8 assay revealed that the overexpression of CSN8 significantly inhibited the proliferation of HCT116 and DLD-1 cells compared to the control cells (Fig. 1m). CSN8 overexpressed HCT116 and DLD-1 cells, which were found to have higher vitality under hypoxia, serum deprivation conditions, or 5-FU treatment compared to the control cells (Figs. 1n–p). The CSN8-overexpressed HCT116 and DLD-1 cells expressed higher levels of dormant markers (NR2F1, DEC2, p27), hypoxia genes (*HIF-1α*, *GLUT1*), and lower levels of Ki67 (Fig. 1q and r), which indicates that CSN8 may be involved in regulating hypoxia-induced dormancy. The overexpression of CSN8 can suppress the expression of c-Myc (Fig. 1q and r). Proto-oncogene c-Myc regulates cell proliferation, cell-cycle progression, and tumor growth. Further, c-Myc inactivation results in tumor dormancy. Suppression of c-Myc protein levels contributes to cancer cell survival under limited oxygen and glucose [8]. Therefore, the downregulation of c-Myc induced by CSN8 might be an important factor contributing to the induction of a dormant state and apoptosis resistance, as well as the promotion of survival under oxygen- and serum-deprived conditions. It was reported that pluripotency-associated genes increased in dormant tumor cells [9]. We also found that CSN8 overexpression could enhance the expression of pluripotency-associated genes (*SOX2* and *SOX9*; Fig. 1r). In addition, a significant increase in the anti-apoptosis gene *BCL-2* and immunosuppressive gene *PD-L1* expression (Fig. 1r) were detected in CSN8-overexpressed cells, which might be associated with enhanced tolerance to environmental stress and immunoescape. The CRC tissue cDNA array showed that the mRNA expression of CSN8 is positively correlated with that of NR2F1 and HIF-1α (Fig. 1s), which suggests that CSN8 participates in regulating hypoxia-induced dormancy. The silencing of CSN8 further verified that CSN8 could act as an important mediator or regulator of EMT and dormancy (Additional file 3: Figs. S1).

### CSN8 is essential for inducing EMT and dormancy under hypoxic environments and for developing stress resistance

**Fig. 2:**
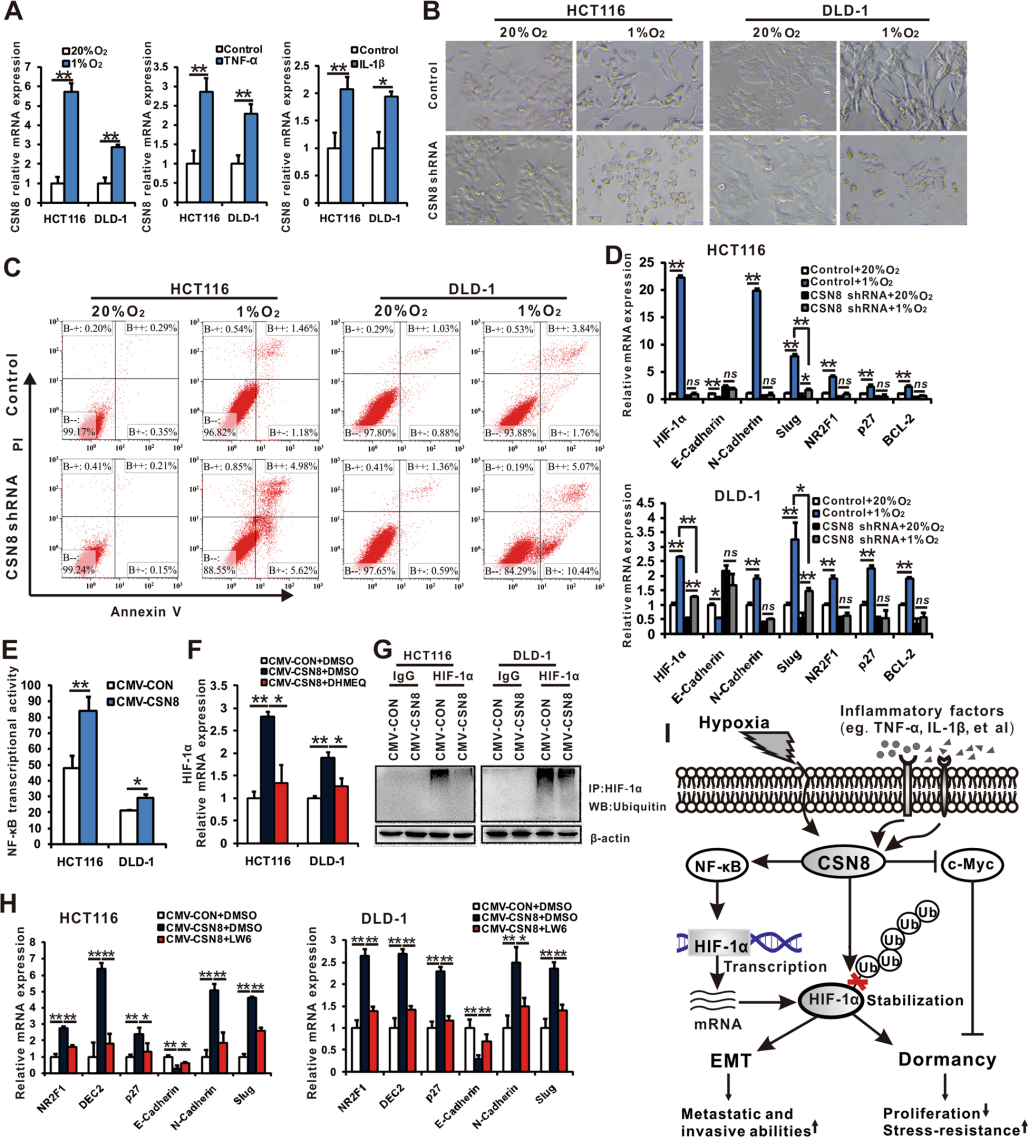
CSN8 is essential for inducing EMT and dormancy under hypoxic environments, and for developing stress resistance. **a** HCT116 and DLD-1 cells were cultured under 20% O_2_ or 1% O_2_ conditions, or treated with TNF-α (20 ng/mL) or IL-1β (20 ng/mL) for 24 h, and then real-time PCR was used to analyze CSN8 mRNA expression. **b** CSN8-silenced CRC cells and control cells were cultured under 20% O_2_ or 1% O_2_ conditions for another 48 h. Photographs were taken using a phase-contrast microscope. **c** Annexin V–FITC/PI staining assay was used to analyze the apoptotic and necrotic cells. **d** Real-time PCR was used to analyze the mRNA expression of the hypoxic response, EMT, and dormancy-related genes. **e** CSN8-overexpressed CRC cells and control cells were transfected with NF-κB dual-luciferase plasmid or control plasmid. After being transfected for 48 h, luciferase activity was detected with the dual-luciferase reporter assay system. **f** CSN8-overexpressed CRC cells were treated with NF-κB inhibitor DHMEQ (25 μM) or DMSO solvent control for 24 h, and then real-time PCR was used to analyze the HIF-1α mRNA levels. **g** Western-blot analysis of the HIF-1α-immunoprecipitated lysates with the anti-ubiquitin antibody. **h** CSN8-overexpressed CRC cells were treated with the HIF-1α inhibitor LW6 (20 μM) or DMSO solvent control for 24 h, and then real-time PCR was used to analyze the mRNA levels of EMT and dormancy-related genes. **i** The model depicts the pivotal role of CSN8 in the EMT and dormancy process of CRC cells under the hypoxic microenvironment. ^*^P < 0.05; ^**^P < 0.01; *ns*, *P ≥* 0.05

To mimic the hypoxic and inflammatory factors of the tumor microenvironment, we cultured HCT116 and DLD-1 cells with 1% oxygen or with the addition of tumor necrosis factor (TNF)-α or interleukin (IL)-1β. We found that CSN8 expression was upregulated under the stimulation of hypoxia, TNF-α, or IL-1β by real-time polymerase chain reaction (PCR) assay (Fig. 2a). To determine whether CSN8 mediated EMT and dormancy induced by hypoxia, we cultured CSN8-silenced HCT116 and DLD-1 cells under 1% oxygen conditions. The results showed that hypoxia failed to drive these two CSN8-silenced CRC cell lines to undergo EMT processes, whereas the control cells obviously demonstrated morphological changes from the more tightly associated epithelial phenotype to the loosely connected, elongated, mesenchymal phenotype (Fig. 2b). Apoptosis assay using Annexin V/propidium iodide (PI) staining and flow cytometry showed that hypoxia significantly induced apoptosis in CSN8-silenced HCT116 and DLD-1 cells when compared to the control cells (Fig. 2c). Real-time PCR analysis revealed that silencing CSN8 inhibited the E/N-Cadherin switch and attenuated increased levels of Slug, NR2F1, p27, HIF-1α, and BCL-2 under hypoxic conditions (Fig. 2d). These results indicate that CSN8 is essential in the EMT and dormancy process in CRC cells, while also contributing to the evasion of apoptosis/necrosis under hypoxic environments. In tumor-bearing mouse models, the tumor formation and tumor growth of CSN8-silenced HCT116 and DLD-1 cells had decreased in comparison with the control cells (Additional file 3: Figs. S2). Silencing of CSN8 might block the dormant process in vivo for CRC cells and impair their adaptive ability in the tumor microenvironment.

As nuclear factor κB (NF-κB) is a direct modulator of HIF-1α expression, we used the luciferase reporter assay to monitor NF-κB activation. CSN8 overexpression significantly enhanced NF-κB transcriptional activation (Fig. 2e). DHMEQ, an NF-κB inhibitor, could suppress the HIF-1α mRNA expression induced by CSN8 overexpression (Fig. 2f). HIF-1α is a short-lived protein and is ubiquitinated and degraded through the von Hippel–Lindau protein (pVHL)–E3 ubiquitin ligase pathway at normoxia [2]. CSN is reportedly associated with deubiquitination activity [6]. The ubiquitination assay showed that CSN8 overexpression could decrease HIF-1α ubiquitination (Fig. 2g). Therefore, these results indicated that CSN8 induced the expression of HIF-1α through NF-κB activation and HIF-1α deubiquitination. Inhibition of HIF-1α by LW6, a HIF-1α inhibitor, resulted in the suppression of the CSN8-mediated E/N-Cadherin switch and the expression of Slug, NR2F1, DEC2, and p27 (Fig. 2h). Hence, CSN8 regulates EMT and dormancy partially by activating the HIF-1α pathway. HIF-1α can bind directly to Sp1, resulting in c-Myc displacement from Sp1 complexes and inhibiting c-Myc transcriptional activity. However, it does not alter c-Myc mRNA and protein levels [10]. Thus, downregulation of c-Myc expression induced by CSN8 may occur independently of the increase in HIF-1α expression, which is an important factor that contributes to dormancy as well.

## Conclusions

Our findings revealed that CSN8 might be a key regulatory molecule that controls the hypoxia-induced EMT and dormancy process, which endows CRC cells with highly aggressive, metastatic and adaptive capacities (Fig. 2i). CSN8 would be an ideal target of disseminated dormant cell elimination and tumor metastasis, recurrence and chemoresistance prevention.

## Supplementary Information

**Additional file 1:.** Supplementary materials and methods.

**Additional file 2: Table S1.** Correlation between the expression of CSN8 and the clinicopathological features of CRC patients**. Table S2.** Correlation between the expression of CSN8 and E-Cadherin. **Table S3.** Primer sequences used for quantitative Real-Time PCR. **Table S4.** Correlation between the expression of CSN8 and the clinicopathological features of CRC patients from a parallel study.

**Additional file 3 Figure S1.** Silencing CSN8 reverses EMT and the dormancy of CRC cells. **Figure S2.** Silencing CSN8 undermines the survival of CRC cells in vivo. **Figure S3.** A parallel tissue microarray assay confirmed CSN8 expression is upregulated in CRC tissues and correlated to poor outcome.

## Data Availability

All the data obtained and/or analyzed during the current study were available from the corresponding authors on reasonable request.

## Abbreviations

CRC: Colorectal cancer
5-Fu: 5-fluorouracil
EMT: Epithelial-mesenchymal transition
HIFs: Hypoxia inducible factors
DTCs: Disseminated tumor cells
MMPs: Matrix metalloproteinases
HNSCC: Head and neck squamous cell carcinoma
CSN: COP9 signalosome
IHC: Immunohistochemistry
NF-κB: Nuclear factor κB
