# Supplementary material for: CSN8 is a key regulator in hypoxia-induced epithelial–mesenchymal transition and dormancy of colorectal cancer cells
Source: Mol Cancer. 2020 Dec 1;19:168. doi: 10.1186/s12943-020-01285-4 (PMC7708218; doi:10.1186/s12943-020-01285-4)
Supplement: Supplementary file 1 — Additional file 1:. Supplementary materials and methods. [file 12943_2020_1285_MOESM1_ESM.docx]

**Materials and Methods**

**Cell lines and cell culture conditions**

Colon cancer cell lines HCT116 and DLD-1 were obtained from the Chinese Academy of Science Cell Bank. Cells were maintained in Roswell Park Memorial Institute (RPMI) 1640 supplemented with 10% fetal bovine serum (FBS). All cells were grown in a humidified atmosphere of 5% CO_2_ at 37°C.

**Quantitative real-time PCR**

Total RNA was extracted using the RNeasy Mini Kit (Qiagen, Venlo, Netherlands) in accordance with the manufacturer’s instructions. Total RNA was subjected to reverse transcription using random hexamers and Superscript II (Thermo Fisher Scientific, Waltham, MA, USA). Quantitative real-time polymerase chain reaction (PCR) assays were performed using SYBR Green Master Mix (Thermo Fisher Scientific), and subsequently run and analyzed using a qTower Real-Time PCR System (Analytik Jena, Jena, Germany).The primer sequences used are listed in (Additional file 2:Table S3). The data were normalized to a β-actin reference. All primers were purchased from Sangon Biotech Inc (Shanghai, China).

**Tissue microarrays (TMAs)**

Primary colorectal cancer samples were used for the construction of a tissue microarray. The samples on the tissue chip (product number: HCol-Ade180Sur-14; Shanghai Biochip Co., Ltd., Shanghai, China) were obtained from 90 colorectal cancer patients with different TNM stages. Matched pairs of 1 mm diameter cylinders from two different areas, the center of the tumor tissue, and the sample adjacent to the tumor, were included in each case to ensure reproducibility and homogenous staining of the slides. Sections of 4 μm thickness were mounted on poly-L-lysine-coated slides for subsequent staining with different anti-human antibodies using a two-step protocol as follows. Briefly, the tissue microarrays were incubated at 63°C in a chamber for 1 hour, deparaffinized with xylene, and rehydrated with a series of ethanol of different concentrations. The slides were boiled in sodium citrate buffer solution (0.01 M, pH 6.0) for 5 minutes and EDTA (0.01 M, PH 9.0) for 20 minutes for antigen retrieval. Following immersion in the endogenous peroxidase blocking solution (38.4 mL of methanol + 12 mL of 30% H_2_O_2_ + 9.6 mL of distilled water) for 15 minutes at room temperature and washed with PBS for three times, the slides were incubated with rabbit anti-CSN8 antibody, rabbit anti-E-Cadherin antibody, or rabbit IgG isotype control (Thermo Fisher Scientific) at 1:400 dilution for 30 minutes at room temperature. Rinsed three times in PBS, the slides were then reacted with the EnVision™ Detection Systems Peroxidase/3’3-diaminobenzidine tetrahydrochloride (DAB), rabbit/mouse (DAKO, Glostrup, Denmark) for 30 minutes at room temperature and visualized by DAB incubation. After being stained with hematoxylin and dehydrated with a series of ethanol of different concentrations, the slides were covered for observation. Immunohistochemical staining was assessed with an Aperio pathological scanning system (Leica, Germany). The staining was scored according to the staining intensity and the distribution of cells stained. Distribution was evaluated as none (0), ≤10% (1), 10%–50% (2), 50%–80% (3), >80% (4). Intensity was evaluated as none (0), faint (1), moderate (2), strong (3), or very strong (4). The final staining scores were calculated as the product of staining intensity multiplied by the percentage of stained cells. Each patient was represented by the mean value from the tumor and the adjacent tissues, respectively.

**qRT-PCR patient tissue arrays**

The qRT-PCR was used to measure CSN8, HIF-1α, and NR2F1 expression levels in colorectal cancer tissue cDNA arrays (product number: cDNA-HColA060CS02; Shanghai Biochip Co., Ltd.) containing 30 matched-pair samples (the tumor tissue and the sample adjacent to the tumor). All samples were normalized to the expression level of the housekeeping gene, β-actin. The comparative ΔΔCt method was used to calculate the normalized relative mRNA expression fold change relative to the sample adjacent to the tumor.

**Lentivirus construction and transfection**

CSN8-shRNA (short hairpin RNA) expression lentiviral particles and control shRNA lentiviral particles were designed and provided by Genomeditech (Shanghai, China). The lentivirus supernatant was transfected into HCT116 and DLD-1 cells with 8 μg/mL of polybrene (Genomeditech) to establish the stable cell lines HCT116-shRNA-CSN8 and DLD-1-shRNA-CSN8. The controls were HCT116-shRNA-control and DLD-1-shRNA-control, respectively.

For the CSN8 overexpression study, the CSN8 cDNA sequences were inserted into the lentiviral expression vector PGMLV-CMV-MCS-EF1-ZsGreen1-T2A-Puro (Genomeditech,) to generate the PGMLV–CSN8 plasmid. For plasmid transfection, HEK-293T cells were seeded in a 10 cm tissue culture plate and incubated at 37°C, 5% CO_2_. When HEK-293T cells achieve 50%–70% confluence the next day, a cocktail was made by putting the constructed plasmid, packaging plasmid, and envelope plasmid to serum-free Opti-MEM, which was added to the HEK-293T cells. The cells were incubated at 37°C, 5% CO_2_ for 24 hours and the media was changed the next day. The lentiviral particles were harvested and stored in an environment –80°C 2 days later. To stably transfect the target cells, the lentiviral particle or control lentiviral particle solution with polybrene was added to the 80%–90% confluent HCT116 or DLD-1 cells. Positive transfected cells were selected by fluorescence-activated cell sorting based on GFP expression.

**Wound healing, Transwell migration, and invasion assays**

For the wound-healing assay, the cell lines were seeded onto 24-well plates. When cell confluence reached ~80%, scratch wounds were made by scraping the cell layer across each culture plate using the tip of a 10 μL pipette. After wounding, the debris was removed by washing the cells with PBS. Wounded cultures were incubated in serum-free medium for 48 hours, and then five fields were randomly picked from each scratch wound and visualized by microscopy to assess cell migration ability. The experiments were performed in triplicate.

Transwell migration assay was performed using 8.0 μm Transwell permeable supports (BD Falcon, San Jose, CA, USA). Then, 100 μL/well (5×10^4^ cells/mL) of the cell suspension was placed onto the upper chambers. The lower chambers of the Transwell were filled with 600 μL of medium containing 20% serum. Cells were incubated at 37°C under 5% CO_2_ for 24 hours, and then non-migrating cells on the upper side of the membrane were removed with a cotton swab. Migrating cells on the lower side of the membrane were fixed with 4% paraformaldehyde (PFA) and stained with crystal violet (Sigma-Aldrich Co., St. Louis, MO, USA). Photomicrographs of five random fields were obtained using a bright-field microscope (Olympus, Tokyo, Japan), and cells were counted to calculate the average number of cells.

For the invasion assay, similar experiments were performed using inserts pre-coated with 1:20 diluted matrigel in serum-free media. Then, 100 μL/well (5×10^4^ cells/mL) of the cell suspension was plated on the matrigel and allowed to invade through the membrane towards the medium containing 20% fetal bovine serum (FBS) in the bottom chamber. Following incubation for 48 hours, cells that did not invade through the membrane, as well as cells and matrigel above the membrane were wiped with cotton swabs; cells below the membrane were fixed in 4% PFA and stained with Crystal Violet (Sigma-Aldrich Co.).

**Western blot**

Cell proteins were extracted using RIPA buffer with a protease/phosphatase inhibitor cocktail (Roche Applied Sciences, Mannheim, Germany). Then, 20 μg of total protein from each sample underwent electrophoresis on a 10% sodium dodecyl sulfate (SDS)-polyacrylamide gel electrophoresis (PAGE) and were transferred onto nitrocellulose membranes (Millipore, Billerica, MA, USA). Membranes were blocked in 5% non-fat milk with TBST for 1 hour at room temperature, and then incubated with rabbit anti-CSN8 antibody (Abcam plc, Cambridge, MA, USA), rabbit anti-NR2F1 antibody (Cell Signaling Technologies, Danvers, MA, USA), rabbit anti-c-Myc antibody (Cell Signaling Technologies), rabbit anti-Slug antibody (Cell Signaling Technologies), rabbit anti-p27 antibody, rabbit anti-Ki67 antibody (Abcam plc), rabbit anti-HIF-1α antibody, and rabbit anti-β-actin antibody (Cell Signaling Technologies) at 4°C overnight. Following this, they were washed for three times and incubated with horseradish peroxidase-conjugated secondary antibodies (1:5,000 dilution; Jackson ImmunoResearch, West Grove, PA, USA) for 20 minutes. Membranes were washed three times in TBST, and detected using the ECL regent (Beyotime Biotechnology, Shanghai, China).

**Ubiquitination assay**

For the ubiquitination assays, cells were treated with MG132 (20 μM) for 6 hours. After removal of the media, the cells were washed twice with ice-cold PBS, then lysed using cell lysis buffer (Beyotime Biotechnology). For immunoprecipitation, cell lysates were incubated with rabbit anti-HIF-1α antibody (Cell Signaling Technologies) at 4°C overnight, followed by precipitation with protein A-Sepharose. For immunoblotting, samples were separated by SDS-PAGE and transferred to polyvinylidene difluoride membranes. The membrane was incubated with the mouse anti-ubiquitin antibody followed by incubation with horseradish peroxidase-conjugated secondary antibodies (1:5,000 dilution; Jackson ImmunoResearch, West Grove, PA, USA) for 20 minutes. Membranes were washed three times in TBST and detected using ECL regent (Beyotime Biotechnology).

**Cell proliferation assay**

The proliferation ability of each cell line was examined using the Cell Counting Kit-8 (CCK-8; Beyotime Biotechnology) according to the manufacturer’s instructions. Briefly, 100 μL of 1×10**^4^** cells were seeded into 96-well plates. Cells were cultured at 37°Cand CCK-8 solution (10 µL) was added into each well 24, 48, and 72 hours later. Following incubation at 37°C for another 1 hour, the OD values at 450 nm were measured using a microplate reader (Bio-Rad, Hercules, CA, USA).

**Cell viability assay**

Cell viability was analyzed by the trypan blue exclusion assay. In brief, cells were seeded at 1×10^5^/mL in 24-well plates overnight and then cultured under hypoxic conditions (1% O_2_) or under serum deprivation, or treated with 5-fluorouracil (5-FU; 20µg/mL). At different time points, the cells were trypsinized and stained with 0.4% (w/v) trypan blue (Sigma-Aldrich Co.). Subsequently, viable cells (unstained) were counted using a hemocytometer.

**Dual-luciferase reporter assay**

Overexpressed CMV-driven-CSN8 and control cells were transfected with NF-κB dual-luciferase plasmid (Beyotime Biotechnology), as well as control plasmid. After transfection for 48 hours, luciferase activity was detected with the dual-luciferase reporter assay system (Beyotime Biotechnology). The relative luciferase signal was presented as firefly luciferase activity normalized to renilla luciferase activity.

**Apoptosis assay**

For the apoptosis assay by flow cytometry, cells were seeded at 1×10^5^/mL in 24-well plates overnight and then cultured under hypoxic conditions (1% O_2_) or treated with 5-FU for 48 hours. Then, the cells were incubated with FITC-Annexin V and propidium iodide (PI) (BD Biosciences, SanJose, CA, USA) in binding buffer for 15 minutes in the dark. Stained cells were immediately analyzed by flow cytometry using the Gallios flow cytometer (Beckman Coulter, Brea, CA,USA).

**Mouse xenograft study**

Nude mice were purchased from the Shanghai Laboratory Animal Center (Shanghai, PR China). All animal protocols were approved by the Institutional Laboratory of Animal Care and Use Committee at Soochow University (Suzhou, PR China). HCT116-shRNA-CSN8 or HCT116-shRNA-control cells (2×10^6^ cells/100µL/site) were injected subcutaneously into the bilateral flanks of 6-week-old nude mice. Then, 2×10^6^/site of DLD-1-shRNA-CSN8 or DLD-1-shRNA-control cells were implanted into the nude mice with the same approach. Tumor growth was monitored every 3 days. Tumor size was assessed by measuring the largest perpendicular diameters, and was recorded as the tumor volume, as follows: V = 1/6π × (length) × (width) × (width). The formed tumor masses were removed and weighed after the tumor-bearing mice were sacrificed.

**Statistical analysis**

Statistical analysis was performed using the SPSS statistical software package (standard version 17.0; IBM Corporation, Armonk, NY, USA). The optimal cut-off level for the high CSN8 expression score or E-Cadherin expression score was determined by the receiver operating characteristics (ROC) curve analysis. For univariate survival analysis, survival curves were obtained using the Kaplan–Meier method. The correlation between CSN8 expression and the clinicopathological features of colorectal cancer patients was analyzed using the chi-squared test. The correlation between CSN8 expression and the expression of E-Cadherin in CRC patients was analyzed by Pearson’s correlation analysis. The independent Student’s *t*-test was performed to analyze the statistical significance between two preselected groups. *P*-values < 0.05 were considered statistically significant.
