# Supplementary material for: CSN8 is a key regulator in hypoxia-induced epithelial–mesenchymal transition and dormancy of colorectal cancer cells
Source: Mol Cancer. 2020 Dec 1;19:168. doi: 10.1186/s12943-020-01285-4 (PMC7708218; doi:10.1186/s12943-020-01285-4)
Supplement: Supplementary file 2 — Additional file 2: Table S1. Correlation between the expression of CSN8 and the clinicopathological features of CRC patients. Table S2. Correlation between the expression of CSN8 and E-Cadherin. Table S3. Primer sequences used for quantitative Real-Time PCR. Table S4. Correlation between the expression of CSN8 and the clinicopathological features of CRC patients from a parallel study. [file 12943_2020_1285_MOESM2_ESM.zip › Additional File 2. Table S1.docx]

**Table S1. Correlation between the expression of CSN8 and the clinicopathological features of CRC patients**

| **Variable** | **All cases** | **Low CSN8** | **High CSN8** | ***P*-value∆** |
| --- | --- | --- | --- | --- |
| **Gender** |  |  |  | 0.745 |
| Male | 45 | 23 | 22 |  |
| Female | 42 | 20 | 22 |  |
| **Age at diagnosis (y)** |  |  |  | 0.326 |
| <65 years | 39 | 17 | 22 |  |
| ≥ 65 years | 48 | 26 | 22 |  |
| **Histological grade** |  |  |  | 0.770 |
| G1 | 20 | 9 | 11 |  |
| G2 | 64 | 32 | 32 |  |
| G3 | 3 | 2 | 1 |  |
| **pT status** |  |  |  | 0.716 |
| T1+T2 | 11 | 6 | 5 |  |
| T3+T4 | 76 | 37 | 39 |  |
| **Lymph node metastasis** |  |  |  | 0.023 |
| Negative | 61 | 35 | 26 |  |
| Positive | 26 | 8 | 18 |  |
| **Clinical stage** |  |  |  | 0.026 |
| I+II | 59 | 34 | 25 |  |
| III+IV | 28 | 9 | 19 |  |

∆, Chi-square test
