# Supplementary material for: CSN8 is a key regulator in hypoxia-induced epithelial–mesenchymal transition and dormancy of colorectal cancer cells
Source: Mol Cancer. 2020 Dec 1;19:168. doi: 10.1186/s12943-020-01285-4 (PMC7708218; doi:10.1186/s12943-020-01285-4)
Supplement: Supplementary file 2 — Additional file 2: Table S1. Correlation between the expression of CSN8 and the clinicopathological features of CRC patients. Table S2. Correlation between the expression of CSN8 and E-Cadherin. Table S3. Primer sequences used for quantitative Real-Time PCR. Table S4. Correlation between the expression of CSN8 and the clinicopathological features of CRC patients from a parallel study. [file 12943_2020_1285_MOESM2_ESM.zip › Additional File 2. Table S2.docx]

**Table S2. Correlation between the expression of CSN8 and E-Cadherin**

|  | CSN8 expression | |  |  |  |
| --- | --- | --- | --- | --- | --- |
|  | Low | High | Total | r | *P*-value* |
| E-cadherin expression |  |  |  | -0.361 | *P*<0.01 |
| Low | 15 | 35 | 50 |  |  |
| High | 28 | 9 | 37 |  |  |
| Total | 43 | 44 | 87 |  |  |

#### r: Pearson’s correlation coefficient

*Correlation is statistically significant at the 0.05 level (two-tailed)
