# Supplementary material for: CSN8 is a key regulator in hypoxia-induced epithelial–mesenchymal transition and dormancy of colorectal cancer cells
Source: Mol Cancer. 2020 Dec 1;19:168. doi: 10.1186/s12943-020-01285-4 (PMC7708218; doi:10.1186/s12943-020-01285-4)
Supplement: Supplementary file 2 — Additional file 2: Table S1. Correlation between the expression of CSN8 and the clinicopathological features of CRC patients. Table S2. Correlation between the expression of CSN8 and E-Cadherin. Table S3. Primer sequences used for quantitative Real-Time PCR. Table S4. Correlation between the expression of CSN8 and the clinicopathological features of CRC patients from a parallel study. [file 12943_2020_1285_MOESM2_ESM.zip › Additional File 2. Table S3.docx]

**Table S3. Primer sequences used for quantitative Real-Time PCR**

| **Gene** | **Forward Primer (5’-3’)** | **Reverse Primer (5’-3’)** |
| --- | --- | --- |
| CSN8 | GCGGAAAGCGCCTTTAGT | AAGCATTCTGATTCTGCCCAT |
| E-Cadherin | TACACTGCCCAGGAGCCAGA | TGGCACCAGTGTCCGGATTA |
| N-Cadherin | CCTGAAGCCAACCTTAACTGAG | CTGTGCTTACTGAATTGTCTTGGG |
| Vimintin | TCAGAGAGAGGAAGCCGAAAAC | GGAGTTTCTTCAAAAAGGCAATC |
| MMP2 | GTCGCCCATCATCAAGTTCC | CATCTTCTTTAGTGTGTCCTTCAGC |
| MMP3 | GCTCCCGAGGTTGGACCTAC | GTTTCACATCTTTTTTGAGGTCGTA |
| MMP9 | CCTGGAGACCTGAGAACCAATC | CCACCCGAGTGTAACCATAGC |
| Snail | AATCGGAAGCCTAACTACAGCG | AGATGAGCATTGGCAGCGA |
| Slug | GCCAAACTACAGCGAACTGGAC | CGCCCCAAAGATGAGGAGTAT |
| NR2F1 | GCCTCAAAGCCATCGTGCTG | CCTCACGTACTCCTCCAGTG |
| DEC2 | CGAGACGACACCAAGGATACC | TTCTGATGCTGTTGCTCGGT |
| p27 | GGTTAGCGGAGCAATGCGCA | AACCGGCATTTGGGGAACCGTC |
| TGF-β2 | ACTTTCTACAGACCCTACTT | GCCATCAATACCTGCAAATC |
| Ki67 | TCCCGCCTGTTTTCTTTCTGAC | CTCTCCAAGGATGATGATGCTTTAC |
| HIF-1α | GAAAACTTGGCAACCTTGGA | ATCTCCGTCCCTCAACCTCT |
| Glut1 | GCGGAATTCAATGCTGATGAT | CAGTTTCGAGAAGCCCATGAG |
| c-Myc | CCCGCTTCTCTGAAAGGCTC | TCGTCGCAGTAGAAATACGGC |
| SOX2 | CCATGCACCGCTACGACG | GACTTGACCACCGAACCCA |
| SOX9 | AGCAGACGCACATCTCCCC | CGTTGACATCGAAGGTCTCGAT |
| BCL-2 | GCGACTCCTGATTCATTGGG | AAGTACAGCATGATCCTCTGTCAAG |
| PD-L1 | AAACAATTAGACCTGGCTG | TCTTACCACTCAGGACTTG |
| β-actin | CTCCATCATGAAGTGTGACG | TGCTTGCTGATCCACATCTG |
