# Supplementary material for: CSN8 is a key regulator in hypoxia-induced epithelial–mesenchymal transition and dormancy of colorectal cancer cells
Source: Mol Cancer. 2020 Dec 1;19:168. doi: 10.1186/s12943-020-01285-4 (PMC7708218; doi:10.1186/s12943-020-01285-4)
Supplement: Supplementary file 2 — Additional file 2: Table S1. Correlation between the expression of CSN8 and the clinicopathological features of CRC patients. Table S2. Correlation between the expression of CSN8 and E-Cadherin. Table S3. Primer sequences used for quantitative Real-Time PCR. Table S4. Correlation between the expression of CSN8 and the clinicopathological features of CRC patients from a parallel study. [file 12943_2020_1285_MOESM2_ESM.zip › Additional File 2. Table S4.docx]

**Table S4. Correlation between the expression of CSN8 and the clinicopathological features of CRC patients** **from a parallel study**

| **Variable** | **All cases** | **Low CSN8** | **High CSN8** | ***P*-value∆** |
| --- | --- | --- | --- | --- |
| **Gender** |  |  |  | 0.920 |
| Male | 40 | 19 | 21 |  |
| Female | 37 | 18 | 19 |  |
| **Age at diagnosis (y)** |  |  |  | 0.793 |
| <65 years | 24 | 11 | 13 |  |
| ≥ 65 years | 53 | 26 | 27 |  |
| **Histological grade** |  |  |  | 0.550 |
| G1 | 3 | 1 | 2 |  |
| G2 | 41 | 22 | 19 |  |
| G3 | 33 | 14 | 19 |  |
| **pT status** |  |  |  | 0.107 |
| T1+T2 | 8 | 6 | 2 |  |
| T3+T4 | 69 | 31 | 38 |  |
| **Lymph node metastasis** |  |  |  | 0.035 |
| Negative | 49 | 28 | 21 |  |
| Positive | 28 | 9 | 19 |  |
| **Clinical stage** |  |  |  | 0.014 |
| I+II | 43 | 26 | 17 |  |
| III+IV | 34 | 11 | 23 |  |
| **MMR status** |  |  |  | 0.773 |
| pMMR | 70 | 34 | 36 |  |
| dMMR | 7 | 3 | 4 |  |

∆, Chi-square test

The tissue microarray (product number: HCol-Ade180Sur-06; Shanghai Biochip Co., Ltd., Shanghai, China) contained cancer tissues and paracancerous tissues from 90 CRC patients with different TNM stages. Thirteen pairs of cancer and paracancerous tissues were excluded as they were severely broken (4 pairs) or due to lack of complete information of clinic pathological features (9 pairs), and the samples from the other 77 CRC patients were further analyzed. The data from tissue microarray (HCol-Ade180Sur-06) confirmed the findings from tissue microarray (HCol-Ade180Sur-14), which indicated that the high expression of CSN8 was significantly associated with lymph node metastasis (*P*=0.035) and clinical stage (*P*=0.014).

To evaluate the microsatellite instability (MSI) status, the expression of mismatch repair (MMR) proteins MLH1, PMS2, MSH2 and MSH6 in the samples on tissue microarray (product number: HCol-Ade180Sur-06) were analyzed by immunohistochemistry. The specimens on tissue microarray were stained with anti-MLH1, anti-PMS2, anti-MSH2, anti-MSH6 antibodies. Protein expression was considered as negative when nuclear staining was absent in the cancer cells whereas present in adjacent non-cancerous tissue used as an internal positive control. Deficient MMR (dMMR) was defined as negative expression of MLH1 and/or PMS2, MSH2 and/or MSH6, PMS2 alone, or MSH6 alone. Proficient MMR (pMMR) was defined as expression of all 4 MMR proteins MLH1, PMS2, MSH2 and MSH6 in cancer cells. There was no significant correlation between the CSN8 expression and MSI/MMR status (*P*=0.773).
