# Supplementary material for: CSN8 is a key regulator in hypoxia-induced epithelial–mesenchymal transition and dormancy of colorectal cancer cells
Source: Mol Cancer. 2020 Dec 1;19:168. doi: 10.1186/s12943-020-01285-4 (PMC7708218; doi:10.1186/s12943-020-01285-4)
Supplement: Supplementary file 3 — Additional file 3 Figure S1. Silencing CSN8 reverses EMT and the dormancy of CRC cells. Figure S2. Silencing CSN8 undermines the survival of CRC cells in vivo. Figure S3. A parallel tissue microarray assay confirmed CSN8 expression is upregulated in CRC tissues and correlated to poor outcome. [file 12943_2020_1285_MOESM3_ESM.zip › Additional File 3. Figure S1.docx]

**Additional File 3. Figure S1**

**
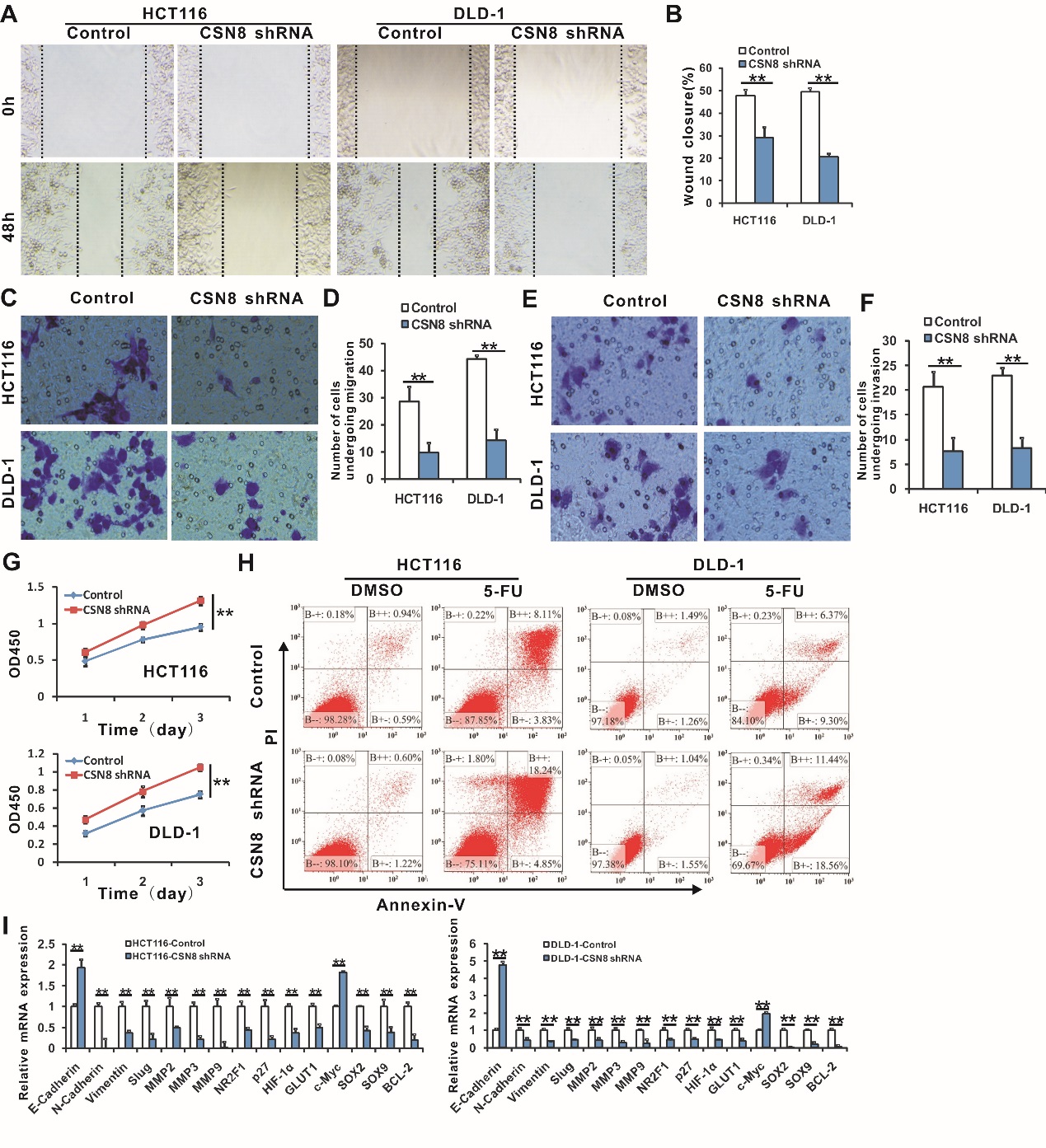
**

**Figure S1. Silencing CSN8 reverses EMT and the dormancy of CRC cells.**

(A, B) Representative scratch–wound images showing the healing ability of CSN8 silenced CRC cells and control cells. (C, D) The migration ability of CRC cells was determined by Transwell migration assay. (E, F) The invasive ability of CRC cells and control cells was analyzed by Matrigel invasion assay. (G) CCK-8 assay analyzed the proliferation activity of CRC cells. (H) The CSN8-silenced CRC cells were treated with 5-FU (20 μg/mL) for another 48 hours, and then Annexin V–FITC/PI staining assay was used for the identification of apoptotic and necrotic cells. (I) Real-time PCR was used to analyze the mRNA expression of the hypoxic response, EMT and dormancy-related genes. Data are presented as the mean ± standard deviation. ^＊^*P*<0.05; ^＊＊^*P*<0.01; *ns*,*P*>0.05.
