# Supplementary material for: CSN8 is a key regulator in hypoxia-induced epithelial–mesenchymal transition and dormancy of colorectal cancer cells
Source: Mol Cancer. 2020 Dec 1;19:168. doi: 10.1186/s12943-020-01285-4 (PMC7708218; doi:10.1186/s12943-020-01285-4)
Supplement: Supplementary file 3 — Additional file 3 Figure S1. Silencing CSN8 reverses EMT and the dormancy of CRC cells. Figure S2. Silencing CSN8 undermines the survival of CRC cells in vivo. Figure S3. A parallel tissue microarray assay confirmed CSN8 expression is upregulated in CRC tissues and correlated to poor outcome. [file 12943_2020_1285_MOESM3_ESM.zip › Additional File 3. Figure S2.docx]

**Additional File 3. Figure S2**


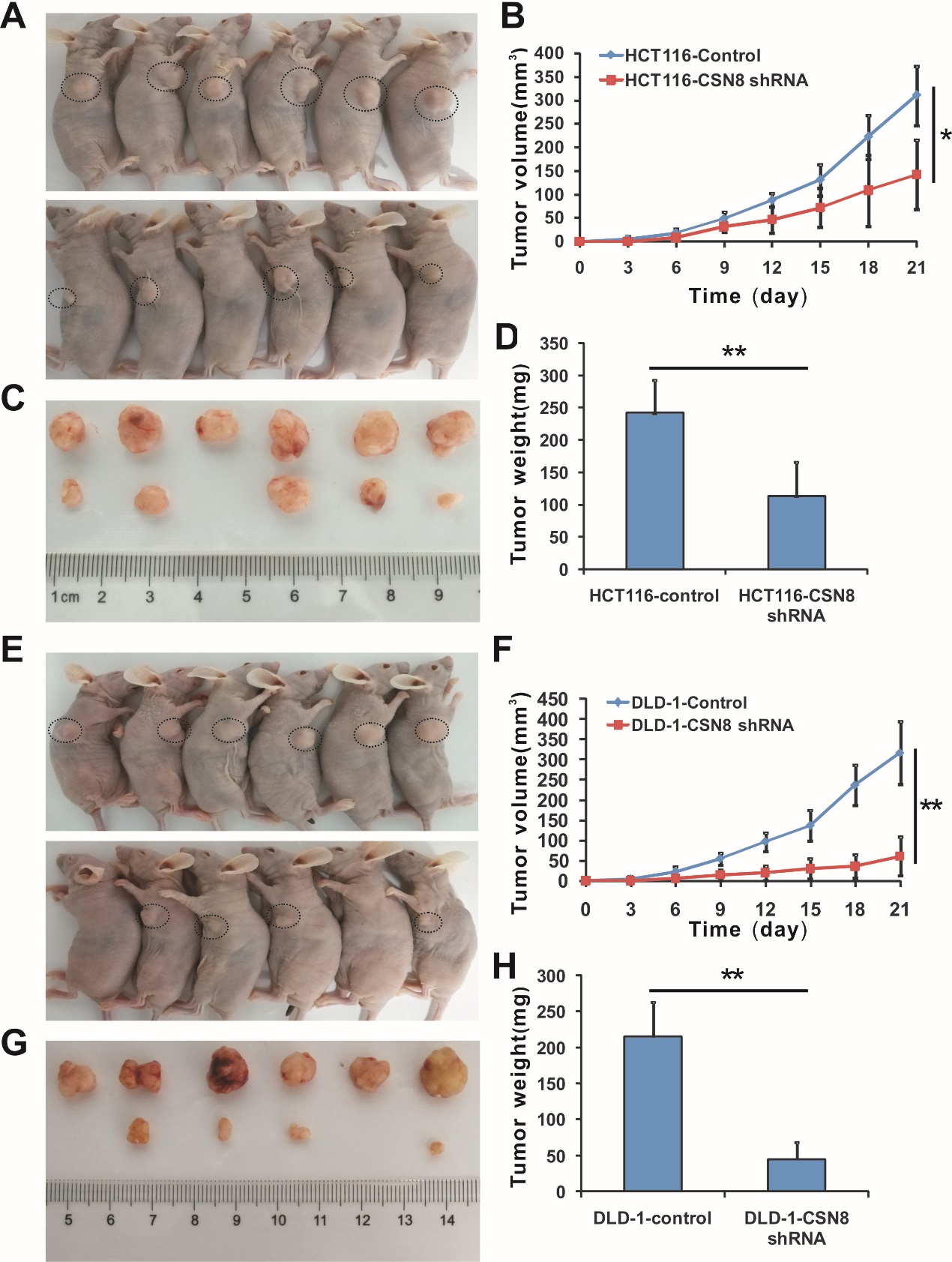


**Figure S2. Silencing CSN8 undermines the survival of CRC cells in vivo.**

(A–D) Silencing CSN8 inhibited the HCT116 cell growth in nude mice. (E–H) Silencing CSN8 decreased DLD-1 cell growth in nude mice. **P*<0.05; ***P*<0.01.
