# Supplementary material for: CSN8 is a key regulator in hypoxia-induced epithelial–mesenchymal transition and dormancy of colorectal cancer cells
Source: Mol Cancer. 2020 Dec 1;19:168. doi: 10.1186/s12943-020-01285-4 (PMC7708218; doi:10.1186/s12943-020-01285-4)
Supplement: Supplementary file 3 — Additional file 3 Figure S1. Silencing CSN8 reverses EMT and the dormancy of CRC cells. Figure S2. Silencing CSN8 undermines the survival of CRC cells in vivo. Figure S3. A parallel tissue microarray assay confirmed CSN8 expression is upregulated in CRC tissues and correlated to poor outcome. [file 12943_2020_1285_MOESM3_ESM.zip › Additional File 3. Figure S3.docx]

**Additional File 3. Figure S3**


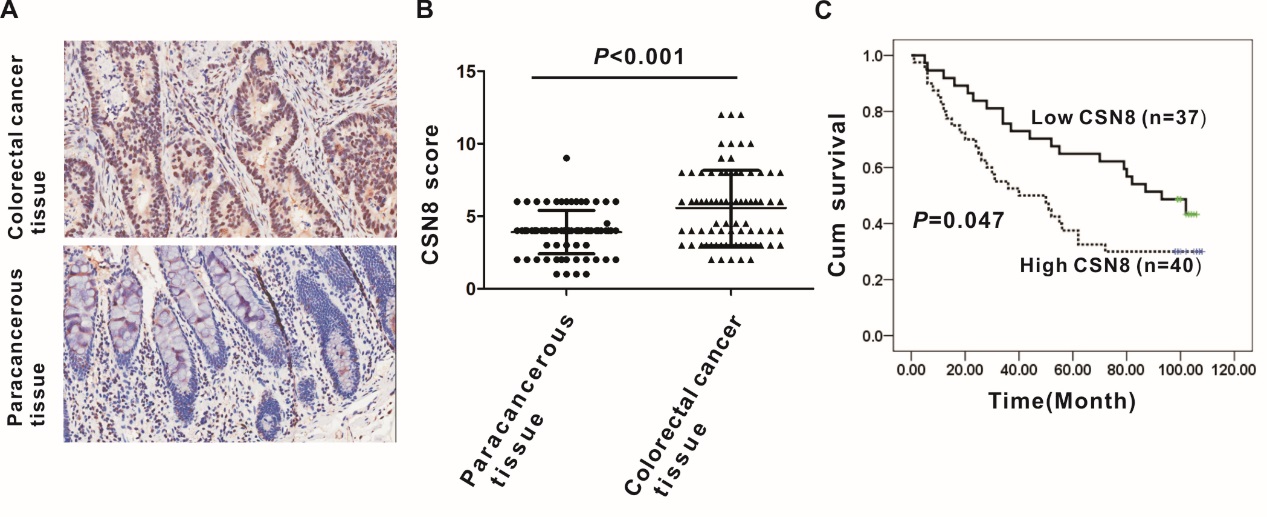


**Figure S3. A parallel tissue microarray assay confirmed CSN8 expression is upregulated in CRC tissues and correlated to poor outcome.**

A tissue microarray assay for the other cohort of tissues from 90 CRC patients (product number: HCol-Ade180Sur-06, Shanghai Biochip Co., Ltd., Shanghai, China) was performed. Thirteen pairs of cancer and paracancerous tissues were excluded as they were severely broken (4 pairs) or due to lack of complete information of clinic pathological features (9 pairs), and the samples from the other 77 CRC patients were further analyzed. (A) Representative immunohistochemistry images showed that CSN8 was dominantly expressed in the nucleus and weakly in the cytoplasm of CRC tissues and adjacent tissues. (B) CSN8 was expressed at significantly higher levels in the nucleus of CRC tissues, as compared to adjacent tissues (*P*<0.001). (C) Kaplan–Meier survival analysis demonstrated that the high expression of CSN8 was significantly associated with poor patient survival (log-rank test, *P*=0.047).
